# Frontiers and hot topics in tumor metabolic reprogramming: a bibliometric analysis from 2014 to 2023

**DOI:** 10.3389/fonc.2025.1570532

**Published:** 2025-06-24

**Authors:** Ping Wen, Guanwen Wang, Ningning Zhang, Qing Shao, Long Wang, Fanli Qu, Xiaohua Zeng

**Affiliations:** ^1^ Chongqing University Cancer Hospital, School of Medicine, Chongqing University, Chongqing, China; ^2^ Department of Breast Cancer Center, Chongqing University Cancer Hospital, Chongqing, China; ^3^ Chongqing Key Laboratory for Intelligent Oncology in Breast Cancer (iCQBC), Chongqing University Cancer Hospital, Chongqing, China

**Keywords:** tumor, metabolic reprogramming, bibliometric, glycolysis, breast cancer

## Abstract

**Introduction:**

Tumor metabolic reprogramming refers to the alteration of metabolic pathways and patterns by tumor cells to adapt to various environmental conditions and energy demands, thereby playing a pivotal role in tumor progression. Targeting tumor metabolism has been shown to exert anti-tumor effects and improve the efficacy of conventional cancer therapies. As a result, tumor metabolic reprogramming has become a research area of considerable clinical value and potential, warranting comprehensive investigation.

**Methods:**

This bibliometric analysis was conducted to systematically summarize the research status, hotspots, and future trends in the field of tumor metabolic reprogramming. Relevant publications from January 1, 2014, to December 31, 2023, were retrieved from the Web of Science Core Collection. A total of 7,311 publications authored by 41,735 researchers from 5,967 institutions in 104 countries/regions were included. Bibliometric analysis and visualization were performed using VOSviewer, CiteSpace, and the bibliometrix R package.

**Results:**

Keyword co-occurrence analysis revealed six major research clusters: tumor microenvironment, various cancers, pathological processes, major mechanisms, epigenetics, and mitochondria. The top three frequently occurring keywords were glycolysis, tumor microenvironment, and mitochondria. China was the most productive country (2,966 publications), followed by Fudan University as the most productive institution (216 publications), and Liu Yang as the most prolific author (40 publications).

**Conclusion:**

Research on tumor metabolic reprogramming has shown rapid global growth and demonstrates significant clinical importance and application potential, especially in the context of breast cancer..

## Introduction

Metabolic reprogramming is a crucial strategy for tumor cells to adapt to the microenvironment and meet the increased energy demands required for rapid proliferation, invasion, and metastasis, playing a pivotal role in tumor progression. Consequently, it provides novel targets and strategies for cancer diagnosis and therapy. Nearly all tumor cells exhibit metabolic abnormalities, and metabolic reprogramming has been recognized as a hallmark of malignancy (1).

Glycolysis, the tumor microenvironment (TME), and mitochondrial metabolism are essential components of tumor metabolic reprogramming. In the 1920s, Otto Warburg discovered that tumor tissue slices *in vitro* consume large amounts of glucose and produce lactate even under aerobic conditions. This phenomenon, known as aerobic glycolysis or the Warburg effect, established the foundation for tumor metabolism research (2). Although this energy production method seems inefficient, it facilitates rapid tumor cell proliferation by providing glycolytic intermediates. The glycolytic pathway is regulated by various oncogenes and tumor suppressor genes, including HIF-1, MYC, p53, and the PI3K/Akt/mTOR pathway (3). A better understanding of the mechanisms regulating aerobic glycolysis could aid in developing glycolysis inhibitors as potential anticancer agents.

The TME and tumor metabolic reprogramming are intricately interconnected. The TME encompasses not only cancer cells but also fibroblasts, immune cells, vascular endothelial cells, stroma, and the extracellular matrix. Interactions between tumor cells and these non-tumor cells govern tumor progression and the TME through the secretion of cytokines, metabolites, and other signaling molecules (4).

In the TME, hypoxia is common, leading to the upregulation of HIF-1α. This, in turn, promotes the expression of glycolytic enzymes and glucose transporters in tumor cells, thereby enhancing glycolysis (5). Furthermore, metabolic reprogramming influences immune cells within the TME, impacting tumor progression. For instance, tumor-associated macrophages often adopt an M2-like metabolic phenotype, which aids tumor cells in evading immune surveillance (6). Additionally, cancer-associated fibroblasts secrete metabolites such as lactate and pyruvate, providing additional nutrient sources for tumor cells and promoting the metabolic reprogramming through metabolic interactions (7).

Tumor metabolic reprogramming significantly impacts the TME. For example, the preference of tumor cells for aerobic glycolysis leads to lactate accumulation, resulting in a lowered pH within the TME. This acidic microenvironment fosters tumor progression while inhibiting the activity of T cells and natural killer cells, facilitating immune evasion (8). Moreover, the aggressive uptake of glutamine by tumor cells limits its availability to immune cells, thereby suppressing the antitumor immune response (9).

Mitochondria are highly dynamic organelles essential for energy metabolism, apoptosis regulation, and cellular signal transduction (10). Due to mutations in oncogenes, tumor suppressor genes, and metabolic enzymes, numerous mitochondrial pathways involved in biomolecule catabolism and energy production are altered in tumors, including the tricarboxylic acid cycle (TCA), oxidative phosphorylation, fatty acid oxidation, glutamine metabolism, and one-carbon metabolism. These alterations lead to metabolic reprogramming that sustains rapid cell proliferation and may result in increased reactive oxygen species (ROS), which cancer cells utilize to maintain pro-tumorigenic signaling pathways. Mitochondria serve as the primary source of ROS, and elevated ROS levels are a common feature of cancer, promoting tumorigenesis by inducing genomic instability, modifying gene expression, and participating in signaling pathways (11, 12).

In recent years, tumor metabolic reprogramming has garnered significant global attention, with targeting abnormal tumor cell metabolism offering new hope for cancer treatment. The volume of literature in this field has steadily increased, however, objective quantitative studies on the current research status, key areas, and future directions remain lacking. Bibliometric analysis, an interdisciplinary field combining mathematics, statistics, and bibliometrics, is used to analyze large volumes of highly heterogeneous literature and objectively display past academic research activities and achievements. This method has become essential for evaluating the quality, credibility, and impact of scholarly works (13, 14). This study conducts a comprehensive bibliometric analysis of selected relevant literature based on the Web of Science Core Collection (WoSCC) database. Statistical analysis were performed on publication trends over the past decade, identifying the most contributive countries, institutions, and authors, and summarizing the top 10 most cited publications in the field. Additionally, relationship networks and hotspots of related literature from 2014 to 2023 were analyzed and visualized. This study reviews the research status, hotspots, progress, and trends in tumor metabolic reprogramming, providing researchers in this field with valuable insights in the following aspects: 1. Publication Trends: How have annual publication in tumor metabolic reprogramming evolved from 2014 to 2023? 2. Key Contributors: Which authors, institutions, and countries are most prolific and influential in this field? 3. Collaborative Networks: What co-authorship and co-institutions networks characterize major collaboration clusters? 4. Thematic Clusters: Which research themes predominate? 5. Future Directions: Based on trend-topic and burst-detection analyses, what emerging topics are likely to drive the field forward?

## Materials and methods

### Searching strategy and data source

WoSCC covers over 21,000 academic journals and is frequently used by researchers, providing the most comprehensive and reliable bibliometric analysis data. In this study, the WoSCC database was the primary source for obtaining data. To minimize potential systematic bias from database updates, a thorough search and screening of relevant publications were conducted on April 24, 2024. All pertinent literature was retrieved and downloaded in “plain text” format.

The literature retrieval and restriction strategies were as follows: 1. The literature was restricted using the TS (“topic,” including title, abstract, and authors’ keywords) keyword search strategy. The search strategy employed was TS = (Neoplas* OR Tumor* OR Cancer* OR Carcinoma* OR Oncology OR Tumor*) AND TS = (Metabolic reprogram* OR Metabolism reprogram*); 2. Publication dates spanned from January 1, 2014, to December 31, 2023; 3. Records available in WoSCC. The article types included in this study were limited to articles or reviews, all written in English.

This search strategy was carefully designed to balance comprehensiveness and relevance. Synonyms and truncations were used to capture a wide range of terms associated with neoplastic diseases and metabolic reprogramming. To verify the adequacy of the search strategy, we performed a manual cross-check of the top 100 most-cited publications in the field to ensure that key landmark studies were included. This approach helped ensure the retrieval of representative and high-impact literature for subsequent bibliometric analysis.

### Data analysis

In this study, R (version 4.3.2) (15), VOSviewer (version 1.16.20) (16), and CiteSpace (version 6.3.R1) (17) were utilized to conduct bibliometric analysis.

VOSviewer was employed for co-authorship and keywords co-occurrence analysis (18).

CiteSpace was used to conduct co-citation analysis of references and identify references experiencing significant citation increases during 2014-2023, revealing the knowledge base and evolution of the field. Results were clustered based on keywords or disciplines, with modularity Q > 0.3 and mean silhouette > 0.5 indicating stable and reliable clustering outcomes (14). The cited references were analyzed using the parameters: time slices (2014–2023), years per slice, node type (references), and g-index as the selection criterion (scale factor k = 25). Additionally, CiteSpace was employed for generating dual-map visualizations of journal connections based on citation and co-citation relationships.

The bibliometrix R package (version 3.2.1) was used to identify highly cited references and create thematic evolution and thematic maps.

All original data used in this study were obtained from publicly available databases and did not involve participant data, thus obviating the need for ethical review.

## Results

### Overview of publication status

**Figure 1:**
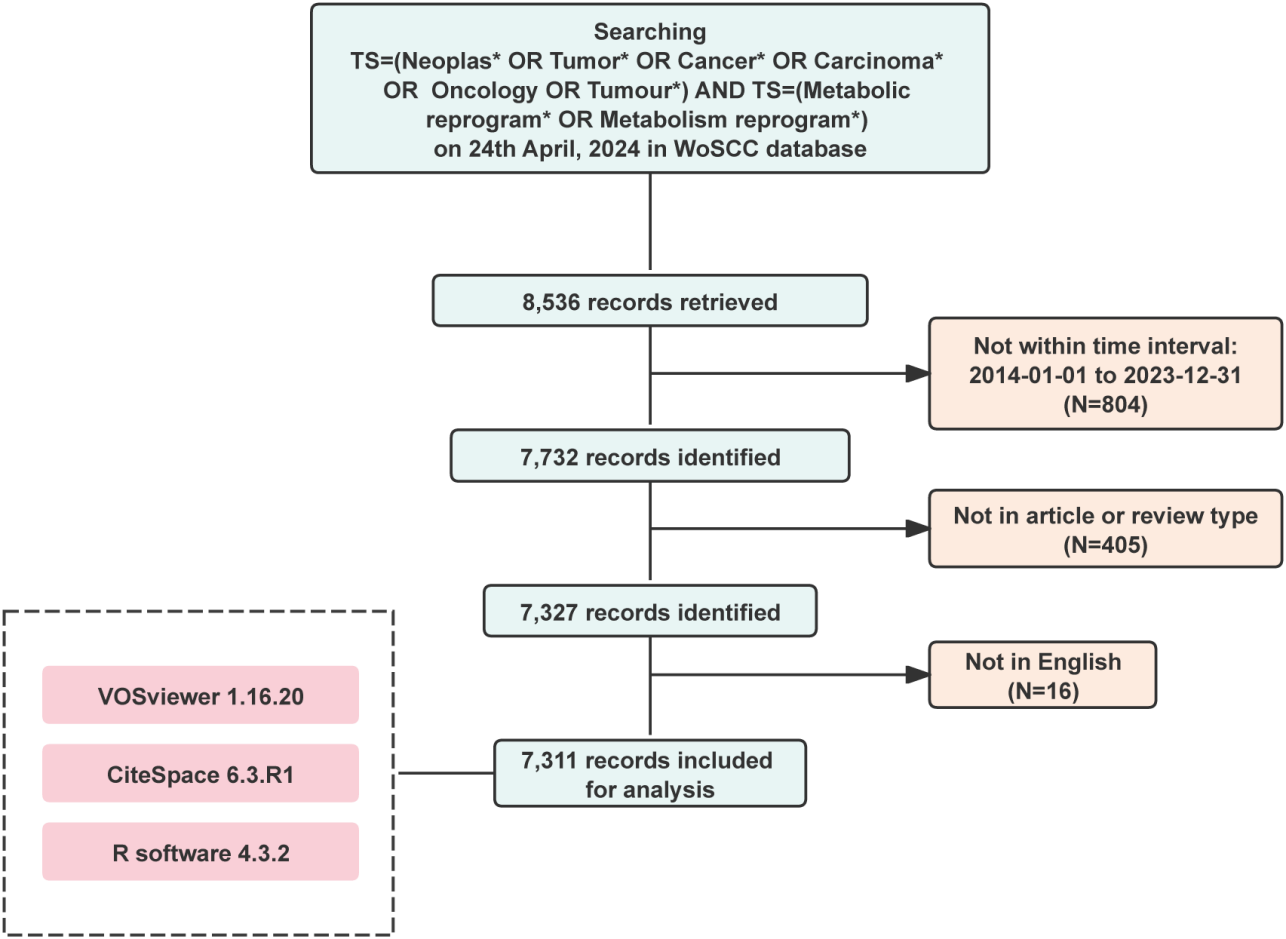
Flowchart of the search strategy and exclusion criteria. The symbol * is a wildcard symbol used to expand the search scope and improve result coverage.

**Figure 2:**
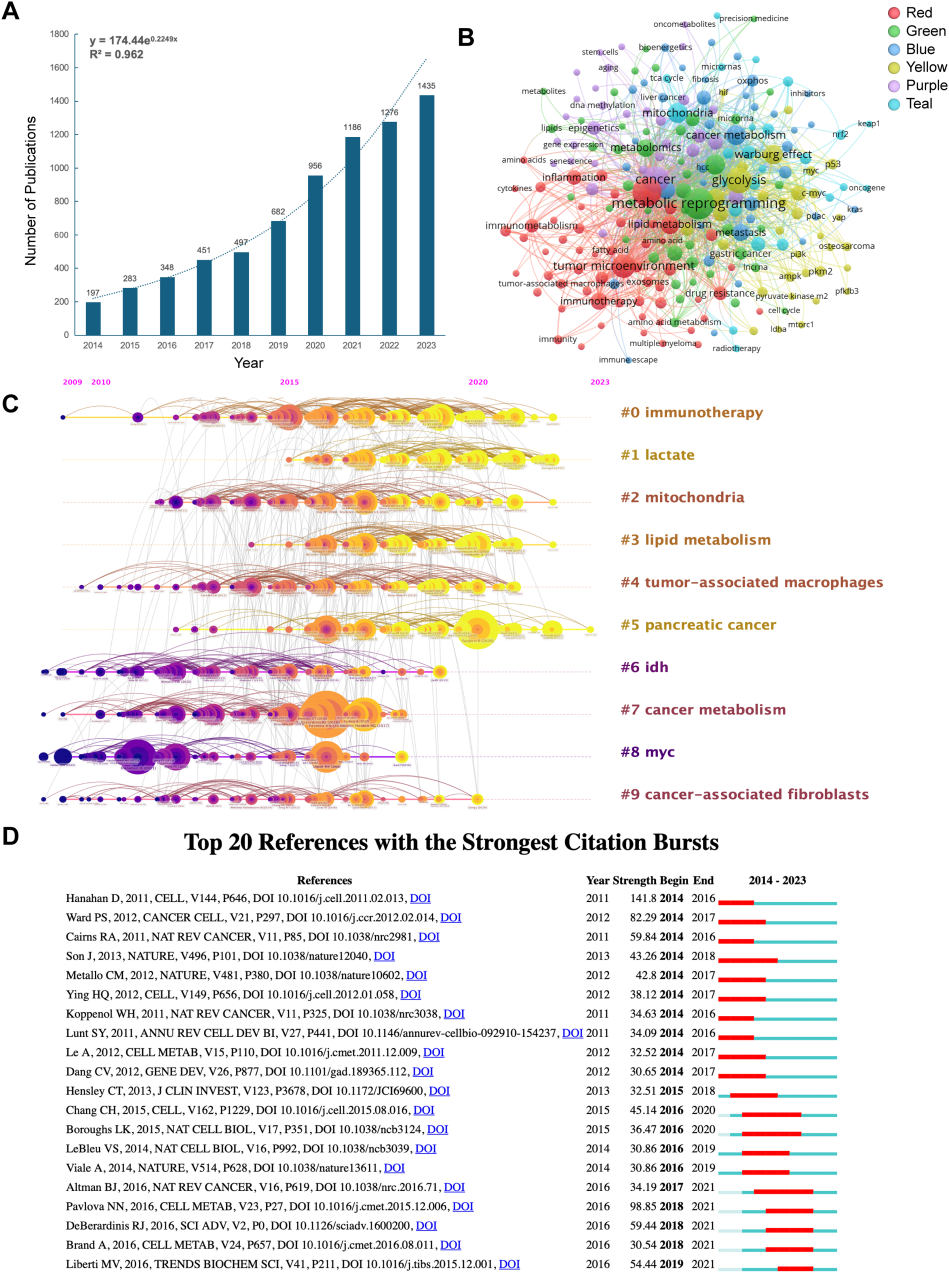
The hotspots and analysis of references co-citation of publications in the field of tumor metabolic reprogramming. **(A)** Trend graph of the growth of annual and cumulative annual number of publications. The dashed curve representing a model fitted trend line to illustrate the overall growth trajectory. **(B)** Authors’ keywords co-occurrence network. **(C)** The co-citation analysis of references in timeline manner. **(D)** The top 20 references with the strongest citation bursts.

A total of 8,536 publications were retrieved from WoSCC, out of which 7,311 were ultimately selected for bibliometric analysis after excluding criteria based on publication time, article type, and language. The flowchart is illustrated in **Figure 1**. Among these, 4,915 articles (67.2%) and 2,396 reviews (32.8%) were included. From 2014 to 2023, there has been a steady increase in the number of publications on tumor metabolic reprogramming, rising from 197 articles in 2014 to 1,435 articles in 2023, marking a growth of 628.4% (**Figure 2A**). The total citation counts for all publications amounted to 263,775, with an average of 36.08 citations per publication.

### Frequency and clustering analysis of keywords

To understand the core content and hotspots in this field, an analysis was conducted on authors’ keywords extracted from 7,311 retrieved publications, yielding 9,830 authors’ keywords. **Table 1** presents the top 20 most frequently occurring authors’ keywords. Key research topics include glycolysis, tumor microenvironment, mitochondria, breast cancer, Warburg effect, lipid metabolism, hypoxia, hepatocellular carcinoma (HCC), metabolomics, and others. Network visualization based on authors’ keywords was performed by VOSviewer, with keyword frequency reflected in node size and total link strength (TLS) reflected in the distance between nodes (19), as shown in **Figure 2B**.

**Table 1:** The top 20 authors’ keywords of tumor metabolic reprogramming.

Rank	Keyword	Records	Total links	Rank	Keyword	Records	Total links
1	Glycolysis	539	1281	11	Immunotherapy	177	430
2	Tumor microenvironment	408	1066	12	Metastasis	173	476
3	Mitochondria	282	680	13	Colorectal cancer	143	295
4	Cancer metabolism	274	571	14	Prognosis	140	316
5	Breast cancer	272	526	15	Aerobic glycolysis	133	327
6	Warburg effect	257	655	16	Inflammation	127	293
7	Lipid metabolism	213	492	17	Glucose metabolism	126	277
8	Hypoxia	201	534	18	Autophagy	122	333
9	Hepatocellular carcinoma	193	357	19	Oxidative phosphorylation	111	328
10	Metabolomics	190	419	20	Pancreatic cancer	110	263

To enhance the readability and clarity of the co-occurrence network, the minimum keyword occurrence threshold was set to 15, resulting in the inclusion of 227 high-frequency keywords. These keywords were grouped into six distinct clusters, each represented by a different color. Notably, the clusters exhibited substantial overlap and interconnection, indicating that research in tumor metabolic reprogramming is integrative rather than fragmented. The red cluster focuses on the interactions between tumor microenvironment and tumor metabolism reprogramming, indicating research emphasis on lipid metabolism, immune metabolism, amino acid metabolism, and inflammation. The green cluster comprises keywords related to various cancer types, including breast cancer, gastric cancer, glioma, and bladder cancer, reflecting the tumor-specific characteristics of metabolic reprogramming and emphasizing the potential for precision targeting based on metabolic vulnerabilities in different cancers. The blue cluster captures key pathological processes influenced by metabolic reprogramming, such as hypoxia, endoplasmic reticulum stress, epithelial-mesenchymal transition, chemotherapy resistance, and immune evasion. These findings underscore the functional roles of metabolic reprogramming in tumor progression. The yellow cluster emphasizes the major mechanisms of tumor metabolism reprogramming, such as the Warburg effect, PI3K, MYC, HIF-1α, AMPK, and p53. The purple cluster underscores the relationship between epigenetics and tumor metabolism reprogramming, focusing on DNA methylation and miRNA. The teal cluster represents the crucial role of mitochondria in tumor metabolism reprogramming, including the TCA cycle and ROS.

### References co-citation, clustering, timeline and bursts

While keyword analysis provides a snapshot of research focuses, reference co-citation analysis offers insight into the intellectual structure and foundational literature of the field. Therefore, we next examined co-citation patterns, cluster timelines, and citation bursts to reveal the development and transformation of key research themes. **Table 2** presents the top 10 most cited publications in the field of tumor metabolic reprogramming, with the review titled “The emerging hallmarks of cancer metabolism” by Pavlova NN et al. being the most cited (N=3,483). This review summarizes research advancements in tumor metabolism, providing a comprehensive overview. Tumorigenesis depends on cell metabolism reprogramming. Various metabolic pathways are autonomously altered by cancer cells to meet increased bioenergetic and biosynthetic demands. Additionally, changes in intracellular and extracellular metabolites associated with cancer-related metabolic reprogramming have profound effects on gene expression, cell differentiation, and the tumor microenvironment. The review categorizes cancer-related metabolic alterations into six features: 1. deregulated uptake of glucose and amino acids; 2. use of opportunistic modes of nutrient acquisition; 3. use of glycolysis/TCA cycle intermediates for biosynthesis and NADPH production; 4. increased demand for nitrogen; 5. alterations in metabolite-driven gene regulation; 6. metabolic interactions with the microenvironment.

**Table 2:** Top 10 publications with high citations in the field of tumor metabolic reprogramming.

Rank	Title	Source	IF (2023)	IF (5 year)	JCR (2023)	Publication year	Total citation	Average citation per year	Type	Doi
1	The emerging hallmarks of cancer metabolism	CELL METABOLISM	27.7	31.2	Q1	2016	3483	387.00	Review	10.1016/j.cmet.2015.12.006
2	The biology and function of fibroblasts in cancer	NATURE REVIEWS CANCER	72.5	77.2	Q1	2016	2574	286.00	Review	10.1038/nrc.2016.73
3	AMPK: guardian of metabolism and mitochondrial homeostasis	NATURE REVIEWS MOLECULAR CELL BIOLOGY	81.3	115.5	Q1	2018	2007	286.71	Review	10.1038/nrm.2017.95
4	Fundamentals of cancer metabolism	SCIENCE ADVANCES	11.7	13.7	Q1	2016	1794	199.33	Review	10.1126/sciadv.1600200
5	Comprehensive and Integrative Genomic Characterization of Hepatocellular Carcinoma	CELL	45.5	49.0	Q1	2017	1260	157.50	Article	10.1016/j.cell.2017.05.046
6	The biology of YAP/TAZ: hippo signaling and beyond	PHYSIOLOGICAL REVIEWS	29.9	45.0	Q1	2014	1181	107.36	Review	10.1152/physrev.00005.2014
7	Defining trained immunity and its role in health and disease	NATURE REVIEWS IMMUNOLOGY	67.7	78.1	Q1	2020	1129	225.80	Review	10.1038/s41577-020-0285-6
8	Metabolic reprogramming in macrophages and dendritic cells in innate immunity	CELL RESEARCH	28.1	36.4	Q1	2015	1063	106.30	Review	10.1038/cr.2015.68
9	Immunometabolism governs dendritic cell and macrophage function	JOURNAL OF EXPERIMENTAL MEDICINE	12.6	14.1	Q1	2016	1030	114.44	Review	10.1084/jem.20151570
10	Metabolic pathways promoting cancer cell survival and growth	NATURE CELL BIOLOGY	17.3	24.2	Q1	2015	1016	101.6	Review	10.1038/ncb3124

IF, Impact Factor; JCR, Journal Citation Reports.

Co-citation analysis of references was conducted and presented in a timeline format (**Figure 2C**). Ten main clusters were identified through co-citation analysis. Nodes placed along the same line in the figure represent a cluster, with labels on the right indicating the cluster’s theme. Node size reflects the frequency of co-citation, with nodes appearing earlier on the left representing classical or relatively established topics, while nodes appearing later on the right represent emerging topics. As depicted in **Figure 2C**, several recent hot topics include immunotherapy, lactate, mitochondria, lipid metabolism, tumor-associated macrophages, and pancreatic cancer. Burst analysis of co-citation reflects periods of rapid changes in citation strength, aiding investigation into the duration of hotness in tumor metabolic reprogramming (**Figure 2D**). A burst is defined as a significant increase in citation received by a publication compared to usual, sustained for at least two years (17). The green line in **Figure 2D** represents the observation period from 2014 to 2023, while the red line indicates the duration of the burst. Notably, the article “Hallmarks of cancer: the next generation” by Hanahan D et al. in 2011 exhibited the highest burst strength (2014-2016, strength 141.8). This review summarized cancer hallmarks, including sustaining proliferative signaling, evading growth suppressors, resisting cell death, enabling replicative immortality, inducing angiogenesis, activating invasion and metastasis, reprogramming of energy metabolism, and evading immune destruction (1). Furthermore, academic works exhibiting high burst strength levels include “The emerging hallmarks of cancer metabolism” by Pavlova NN et al. (2018-2021, strength 98.85) and “Metabolic reprogramming: a cancer hallmark even warburg did not anticipate” by Ward PS et al. (2014-2017, strength 82.29).

### Trends of themes

**Figure 3:**
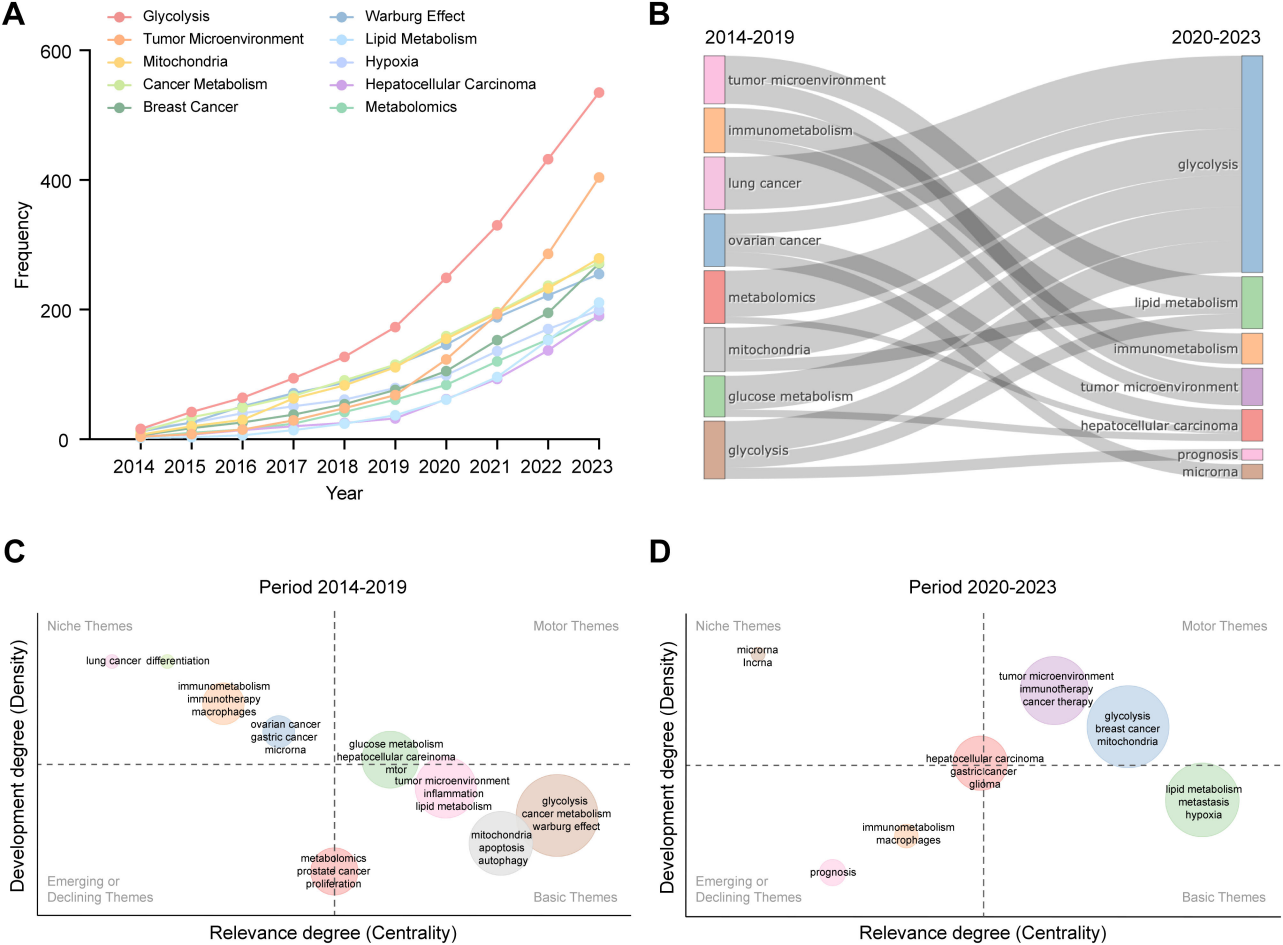
The evolutionary trends of publications in the field of tumor metabolic reprogramming. **(A)** The changes of high-frequency keywords over time. **(B)** Sankey diagram illustrating the thematic evolution of publication themes over the past decade. **(C, D)** Thematic maps during 2014–2019 and 2020–2023.

To complement co-citation findings and further explore the evolution of thematic priorities, we conducted a temporal and structural analysis of keyword trends, enabling a more dynamic understanding of how major topics have shifted over time. First, high-frequency keywords and their temporal changes were analyzed (**Figure 3A**). Next, a Sankey diagram (**Figure 3B**) was used to visualize the evolution of topics from 2014 to 2019 and from 2020 to 2023. Thematic maps based on centrality and density were plotted to illustrate trends in themes during different periods (**Figures 3C, D**). The thematic maps were created based on authors’ keywords, with the horizontal axis representing centrality, indicating the relevance of a theme to the field. The vertical axis represents density, reflecting the degree of development of the theme within the field. Accordingly, four types of themes were identified: niche themes, motor themes, emerging or declining themes, and basic themes. Niche themes are well-developed but have limited relevance to the current field; motor themes are both important and developed, representing cutting-edge topics in the research field; emerging or declining themes are underdeveloped with low internal and external connections, indicating emerging or declining trends; basic themes are considered core and transversal topics in the field (20).

When comparing the periods of 2014–2019 and 2020-2023, a decrease in both centrality and density was observed for the themes “immune metabolism” and “macrophages.” However, the theme “immunotherapy” showed a significant increase in both centrality and density, and it entered motor themes, indicating it is important and well-developed. Additionally, the density of the themes “microRNA,” “tumor microenvironment,” “glycolysis,” and “mitochondria” increased. Notably, a new theme with high centrality and density emerged from 2020 to 2023, namely “breast cancer,” highlighting its rapid development and emerging status, suggesting that metabolic reprogramming may hold significant importance in breast cancer.

### Situation of countries/regions and institutions

**Figure 4:**
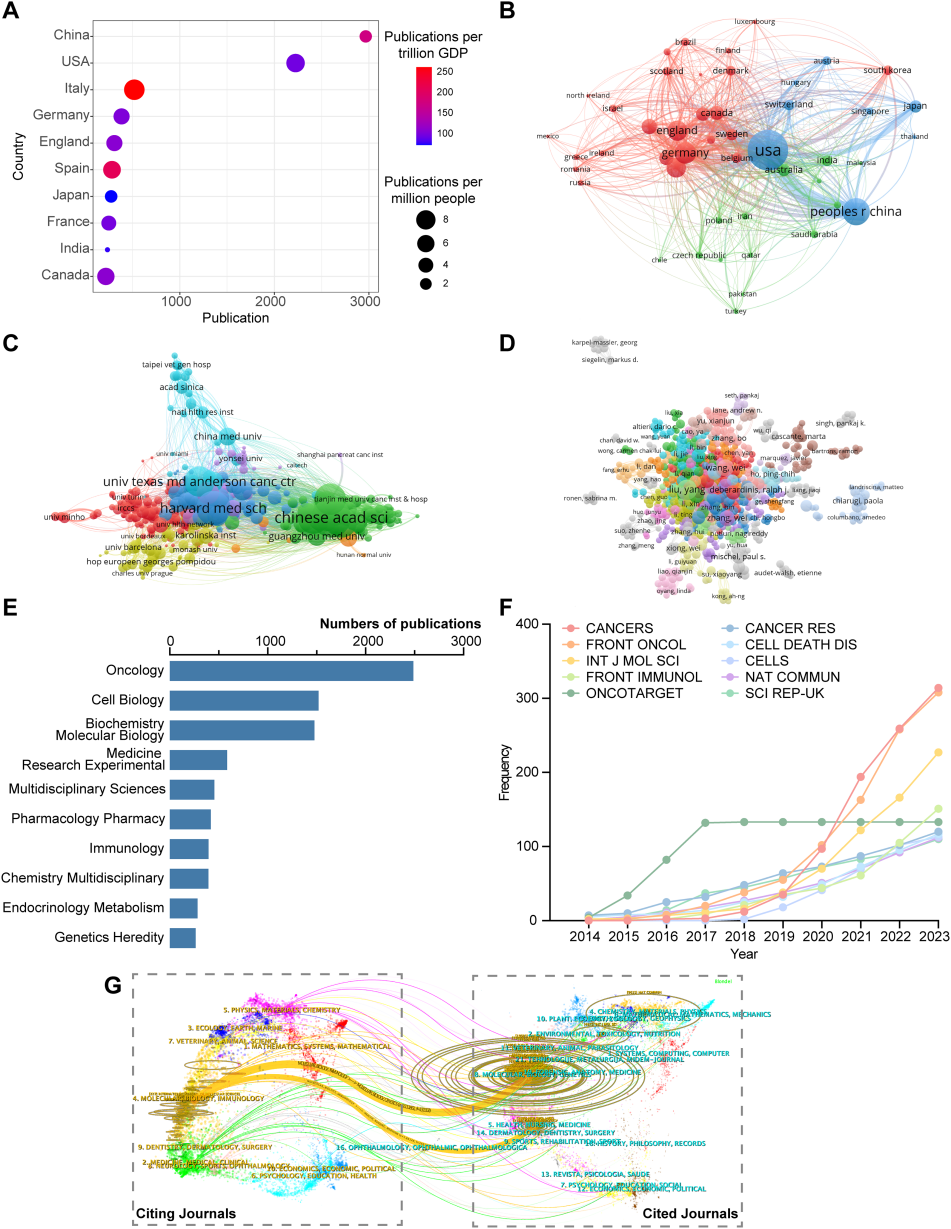
The distribution of publications and the collaborative relationships among different countries/regions, institutions, and authors. **(A)** The top 10 productive countries/regions in this field. **(B-D)** The co-authorship relationships for countries/regions **(B)**, institutions **(C)** and authors **(D)**, respectively. **(E)** The distribution of publications across disciplines. **(F)** The cumulative publication growth patterns of the top 10 most productive journals. **(G)** The dual-map overlay of journals.

Beyond research themes, it is also critical to examine the global distribution of scholarly contributions. We therefore analyzed the geographic and institutional landscape to assess the output, impact, and collaboration patterns across countries and institutions. A total of 104 countries/regions have contributed to the field of tumor metabolic reprogramming. Publication information categorized by country/region was collected and analyzed (**Figure 4A**, **Table 3**). China made the largest contribution, with a total of 2,966 publications. Following China were the United States (2,224 publications), Italy (519 publications), Germany (384 publications), and the United Kingdom (308 publications). Italy had the highest number of publications per trillion GDP (N = 253.20). In terms of citation counts, the United States ranked highest with 122,543 citations, followed by China with 74,819 citations, and Italy with 20,194 citations. Despite having the highest number of publications, China’s average citations per paper (N = 25.23) were significantly lower than those of the United States (N = 55.10) and Italy (N = 38.91).

**Table 3:** The top 10 productive countries/regions in the field of tumor metabolic reprogramming.

Rank	Country/region	Numbers of publications	Publications per million people*	Publications per trillion GDP*	Numbers of citations	Average citations per publication	Centrality	Co-authorship total link strength
1	PEOPLES R CHINA	2966	2.10	165.12	74819	25.23	0.21	790
2	USA	2224	6.67	87.42	122543	55.10	0.43	1632
3	ITALY	519	8.81	253.20	20194	38.91	0.12	442
4	GERMANY	384	4.58	94.06	18313	47.69	0.13	506
5	ENGLAND	308	4.60	99.71	15188	49.31	0.12	447
6	SPAIN	283	5.92	199.60	10530	37.21	0.08	287
7	JAPAN	274	2.19	64.37	11975	43.70	0.05	198
8	FRANCE	249	3.66	89.60	11176	44.88	0.07	254
9	INDIA	235	0.17	68.78	4386	18.66	0.04	162
10	CANADA	218	5.60	100.86	10471	48.03	0.06	236

*Calculations based on 2022 population and GDP data from world bank (https://databank.worldbank.org/). GDP is calculated using GDP (current US$).

A co-authorship analysis was conducted on publications from various countries, revealing that 48 countries have published 15 or more articles. Network mapping analysis was performed to identify and illustrate cooperation relationships among countries/regions (**Figure 4B**). The node centrality indicates the degree of collaboration and communication among countries/regions, with a centrality greater than 0.1 generally indicating denser connections and higher relevance (21). Among them, the United States had a centrality of 0.43, indicating its role as a bridge in paper collaboration and communication across countries. China (N = 0.21), Italy (N = 0.12), Germany (N = 0.13), and the United Kingdom (N = 0.12) were all important nodes in the clusters, representing high levels of cooperation. Additionally, these countries/regions were divided into three colored clusters, with the thickness of connecting lines representing the TLS. The top three countries/regions with the highest TLS were the United States (TLS = 1,632), China (TLS = 790), and Germany (TLS = 506). The blue cluster centered on China and the United States included several countries/regions such as Japan, Thailand, Singapore, Austria, Hungary, and Switzerland. The red cluster comprised most countries, totaling 25 countries, with Germany and the United Kingdom as the focal points. The green cluster centered on Australia and India.

To explore the contributions of institutions to tumor metabolic reprogramming, the number of publications from different institutions was analyzed. Research in this field involved 5,967 institutions worldwide. **Table 4** lists the top 10 productive institutions. Fudan University, Shanghai Jiao Tong University, and Sun Yat-sen University were the top three institutions with the highest number of publications, with 216, 214, and 213 publications respectively. Additionally, eight out of the top 10 institutions were from China, demonstrating a high level of interest and significant contribution from Chinese institutions to research in tumor metabolic reprogramming. The University of Texas MD Anderson Cancer Center received the most citations (N = 11,213) and had the highest average citations (N = 81.25). To further investigate collaboration among institutions, co-authorship analysis was conducted on all publications, revealing seven clusters of institutional cooperation, as shown in **Figure 4C**. The institution with the highest TLS was the Chinese Academy of Sciences (TLS = 437).

**Table 4:** The top 10 productive institutions in the field of tumor metabolic reprogramming.

Rank	Institution	Country/region	Numbers of publications	Numbers of citations	Average citations per publication	Co-authorship total link strength
1	Fudan University	China	216	6615	30.63	307
2	Shanghai jiao tong University	China	214	5928	27.70	279
3	Sun yat-sen University	China	213	6250	29.34	295
4	Chinese Academy of Sciences	China	191	7948	41.61	437
5	Zhejiang University	China	140	4018	28.70	188
6	The University of Texas MD Anderson Cancer Center	USA	138	11213	81.25	287
7	Harvard Medical School	USA	119	8415	70.71	348
8	Huazhong University of Science and Technology	China	117	3998	34.17	128
9	Zhengzhou University	China	116	2113	18.22	149
10	Central South University	China	114	2610	22.89	120

These findings collectively suggest that while China leads in research volume, the United States continues to serve as the central axis of international collaboration and scientific influence. The global network is characterized by both regional clustering and cross-border cooperation, shaping a collaborative ecosystem that is vital for advancing research in tumor metabolic reprogramming.

### Authors and co-authors analysis

At the individual level, author-specific analyses shed light on the key contributors driving this research area. Thus, we further investigated the productivity, impact, and collaboration networks of influential authors. A total of 41,735 authors participated in research on tumor metabolic reprogramming. **Table 5** presents information on the top 10 most productive authors, including their publication counts, citation numbers, and H-index values to provide a comprehensive assessment of their scientific impact. DeBerardinis Ralph J., affiliated with the University of Texas Southwestern Medical Center, was identified as the most cited author (5,548 citations) with an H-index of 23, while Liu Yang, from Chinese Academy of Medical Sciences, was the most prolific author, publishing 40 articles with an H-index of 29. Collaboration among scholars is depicted in **Figure 4D**, where 882 authors who published 5 or more articles were clustered into 30 groups. It was observed that some dispersed clusters lacked collaboration with larger clusters, indicating insufficient cooperation among different research groups.

**Table 5:** The top 10 productive authors in the field of tumor metabolic reprogramming.

Rank	Author	Numbers of publications	Numbers of citations	Average citations per publication	Co-authorship total link strength	H-index	G-index
1	Liu, yang	40	1442	36.05	307	29	52
2	Wang, wei	29	1216	41.93	279	17	33
3	Zhang, wei	27	615	22.78	295	14	30
4	Deberardinis, ralph j.	25	5548	221.92	437	23	28
5	Wang, lei	24	377	15.71	188	24	40
6	Zhang, bo	23	426	18.52	287	17	37
7	Zhang, yi	22	628	28.55	348	26	52
8	Zhang, li	22	335	15.23	128	24	43
9	Mischel, paul s.	21	976	46.48	149	16	21
10	Xu, guowang	20	586	29.30	120	12	21

### Distribution across disciplines and journals

Finally, we assessed the disciplinary distribution and journal landscape to identify the main publication outlets and interdisciplinary nature of research in tumor metabolic reprogramming. Statistical analysis was conducted on the top 10 subject categories defined by WoS classification in the publications (**Figure 4E**). The three primary subject categories in this field were Oncology, Cell Biology, and Biochemistry Molecular Biology, accounting for approximately 75% of the publications. A total of 1,130 academic journals were actively engaged in research on tumor metabolic reprogramming. The cumulative growth patterns of annual publications and information on the top 10 productive journals are presented in **Figure 4F** and **Supplementary Table 1**, respectively. The journal with the highest number of publications was *CANCERS* (N = 314), followed by *FRONTIERS IN ONCOLOGY* (N = 308) and *INTERNATIONAL JOURNAL OF MOLECULAR SCIENCES* (N = 227).

The overlay of journal dual-maps accurately captures the dynamics and trends of disciplinary development, researches on tumor metabolic reprogramming are related to multiple disciplines. Labels on the left side of the map represent citing journals, reflecting the forefront of knowledge, while labels on the right side represent cited journals, reflecting the knowledge foundation. Curves connecting the left and right parts of the map represent citation links, visualizing the relationships between citing and cited journals. As depicted in **Figure 4G**, citing journals are mainly distributed in the fields of molecular, biology and immunology, while cited journals are predominantly distributed in the fields of health, nursing, medicine, molecular, biology, and genetics.

## Discussion

In recent years, tumor metabolic reprogramming has attracted considerable attention due to its critical role in tumor biology and its potential clinical applications. This study conducts a bibliometric analysis of literature published in the field of tumor metabolic reprogramming from 2014 to 2023. The aim is to comprehensively identify research hotspots and development trends, uncover potential innovative pathways, and provide valuable references and guidance for future research.

Over the past decade, the number of publications related to tumor metabolic reprogramming has shown a continuous upward trend, with significant increases in 2017, 2019, 2020, and 2021. This surge is likely associated with key technological advancements and clinical breakthroughs in the field. In 1999, Nicholson et al. introduced the concept of Metabolomics (22). In recent years, the refinement of metabolomics and radiotracer technologies has greatly expanded their application in tumor metabolism research, significantly enhancing the precision and scope of studies (23). Between 2010 and 2016, multiple studies confirmed the anticancer effects of the metabolic regulator metformin in adjunct therapy through modulation of glucose metabolism, further promoting the application of metabolic intervention strategies (24–28). Additionally, the M13–982 study demonstrated that Venetoclax (BCL-2 inhibitor) showed superior efficacy and tolerance compared to ibrutinib in treating relapsed or refractory chronic lymphocytic leukemia (CLL) patients, including those with 17p gene deletions. This led to its accelerated approval by the Food and Drug Administration (FDA) in 2016 for CLL treatment, marking a significant breakthrough in tumor metabolism-targeted therapy. In relapsed or refractory acute myeloid leukemia (AML) patients, an isocitrate dehydrogenase (IDH) 2 inhibitor, Enasidenib monotherapy was well-tolerated, induced hematologic remission, and was associated with a median survival of over 9 months (29). Enasidenib received FDA approval in 2017 for the treatment of IDH2-mutant relapsed or refractory AML, further validating the clinical value of IDH mutation-targeted therapies. In molecularly defined high-risk relapsed or refractory AML patients, Ivosidenib (IDH1 inhibitor) monotherapy exhibited a lower incidence of grade 3 or higher treatment-related adverse events compared to previous outcomes, and induced deep and durable remissions, leading to favorable clinical outcomes (30). Ivosidenib was approved by the FDA in 2018 for the treatment of IDH1-mutant relapsed or refractory AML.

These key events have not only fueled research enthusiasm in the field of tumor metabolic reprogramming but also provided essential scientific and clinical support for developing new anticancer therapies. With the progress of technology and the development of new metabolic targeted drugs, the field of tumor metabolic reprogramming is expected to make greater breakthroughs and provide more innovative strategies for tumor treatment in the future.

Glycolysis is currently a major research focus in tumor metabolic reprogramming. The Warburg effect during tumorigenesis is one of the most classic and extensively studied models of cell metabolic reprogramming. Most malignant cells are highly sensitive to glucose deprivation, reflecting their unique metabolic demands and vulnerabilities. When glucose supply is insufficient, the energy supply to tumor cells rapidly diminishes, leading to impaired cell function and death. Thus, the Warburg effect is a highly attractive therapeutic target (31).

The first medical application based on the Warburg effect was ^18^F-fluorodeoxyglucose. This technique exploits the increased uptake of glucose and ^18^FDG in most cancers compared to normal tissues and was applied clinically in the 1980s (32). It assists in tumor staging, diagnosis, and treatment response evaluation (33). Many glycolysis-targeting drugs have shown promising anticancer effects in preclinical trials. For instance, the glucose transporter type 1 (GLUT1) inhibitor WZB117 blocks glucose entry into cancer cells, disrupting glycolysis, reducing intracellular ATP and glycolytic enzymes, ultimately causing cell cycle arrest, senescence, and necrosis (3, 34). Similarly, the pyruvate analogue 3-bromopyruvate binds to GAPDH in cancer cells, inhibiting its enzymatic function, causing ATP depletion, and leading to apoptosis (35–37). The lactate dehydrogenase (LDH) inhibitor FX11 increases oxidative stress by depleting intracellular ATP, resulting in tumor cell death (38).

Despite promising preclinical results, glycolysis inhibitors have not met clinical expectations. Hexokinase II (HKII) is an enzyme crucial for converting glucose to glucose-6-phosphate in the first step of glycolysis. Lonidamine, an HKII inhibitor, has completed phase III clinical trials. However, its clinical use is limited by pancreatic and liver toxicity (39). Similarly, another glucose analogue, 2-deoxyglucose (2-DG), has shown promising anticancer effects in preclinical models (40–43). Nonetheless, studies have indicated that 2-DG can enhance cancer cell survival (44), and hypoxic cells are prone to 2-DG resistance (45). Therefore, the success of 2-DG as a single-agent anti-glycolysis treatment is challenged. Since metabolic processes are largely common between tumor and normal cells, targeting tumor metabolism can also interfere with normal cells. Metabolic drugs often have narrow therapeutic windows and may cause significant toxicity. This has resulted in less satisfactory outcomes in using tumor metabolism targeting for cancer therapy (46). Therefore, the development of such targeted drugs requires high precision, which remains a major challenge for clinical translation.

In the thematic map, breast cancer has emerged as a rapidly developing topic in recent years, drawing significant attention. Different subtypes of breast cancer are associated with varying activities of oncogenes, tumor suppressor genes, transcription factors, and signaling cascades, leading to distinct metabolic characteristics and dependencies. Luminal A breast cancer often exhibits high levels of monocarboxylate transporter (MCT) 1, LDHB, and glutamine synthetase, resulting in reduced lactate secretion and enhanced glutamine synthesis (47, 48). Basal-like breast cancer typically demonstrates a Warburg-like phenotype, characterized by elevated GLUT1, MCT4, and LDHA expression (48), which increases glucose uptake and lactate secretion. HER2-enriched breast cancer frequently exhibits a glycolytic phenotype consistent with upregulation of the PI3K/AKT-mTOR signaling pathway and functional loss mutations in TP53 (49–51). This subtype also enhances lipid metabolism by increasing the expression and activity of fatty acid-related genes (52). While specific metabolic dependencies are often observed in different breast cancer subtypes, varying metabolic characteristics can also be found within the same subtype due to the complexity of cellular metabolic reprogramming and its interaction with multiple internal and external factors.

Significantly, targeting the metabolic differences between tumor cells and normal cells presents a promising new anticancer strategy. Numerous preclinical studies and clinical trials have confirmed its significant role in breast cancer treatment. Recently, small molecules such as BPTEs, DON, UPGLO0004, and CB-839 have been reported as glutaminase inhibitors with properties like permeability, microsomal instability, and oral bioavailability (53). Among these, CB-839 is an effective selective inhibitor that demonstrates significant antiproliferative activity in triple-negative breast cancer (TNBC) and may have a synergistic effect with chemotherapeutic agents such as paclitaxel (54). Further studies have shown that CB-839, in combination with mTOR inhibitors like everolimus, exerts synergistic inhibitory effects by altering the mTOR pathway (55). Metformin and NF-κB inhibitors mediate glucose uptake and glycolysis while blocking excess lactate export by inhibiting MCT4, leading to metabolic crises within cancer cells. Clinical trials targeting metabolic reprogramming in breast cancer treatment are gradually being conducted. A clinical trial (NCT00096707) for advanced breast cancer patients indicated that combining 2-DG with other anticancer drugs enhances therapeutic efficacy and slows tumor growth (56). Additionally, another clinical trial (NCT03057600) demonstrated that CB-839 combined with paclitaxel shows promising results in treating TNBC. These developments highlight the potential of metabolic reprogramming in advancing breast cancer therapy.

An analysis of the results shows that from 2014 to 2023, 1,705 relevant papers were published in the top ten most productive academic journals in the field of tumor metabolic reprogramming, accounting for 23.32% of the total publications. *CANCERS* ranked first in the number of publications, followed by *FRONTIERS IN ONCOLOGY* and the *INTERNATIONAL JOURNAL OF MOLECULAR SCIENCES*, indicating these journals’ strong interest in tumor metabolic reprogramming research. *NATURE COMMUNICATIONS* has the highest impact factor and the highest average citations per paper, reflecting its high publication quality and significant influence in this field.

These journals are mostly related to oncology and cell biology. The overlay of dual maps shows similar results, indicating the great clinical translational value of research in tumor metabolic reprogramming. Among the top ten most cited articles, only one is an original research article, while the others are reviews, highlighting a milestone discovery. This article, published by the Cancer Genome Atlas Research Network in Cell in 2017, analyzed 363 HCC cases using whole-exome sequencing and DNA copy number analysis, and performed DNA methylation, RNA, miRNA, and proteomics expression analysis on 196 HCC cases. Significant alterations by mutation or downregulation by hypermethylation in genes (ALB, APOB, and CPS1) were observed, potentially leading to metabolic reprogramming in HCC. The progression of hepatocytes to malignant HCC cells likely involves metabolic reprogramming through genetic (ALB, APOB), epigenetic (CPS1), or other mechanisms, transforming cells that support normal physiological functions into those that only support their own growth and division needs (57). This finding surprised researchers and greatly promoted the progress in tumor metabolic reprogramming research.

This study primarily employs bibliometric analysis to investigate the development trajectory, status, and research hotspots in tumor metabolic reprogramming. Primarily, by systematically mapping the evolution of research themes (for example, the rapid emergence of hotspots such as immunotherapy and lactate metabolism), it helps researchers identify underexplored areas and define future research directions. Additionally, the construction of author and institutional collaboration maps, based on co-citation and collaboration networks, provides valuable insights to facilitate interdisciplinary and cross-regional partnerships. Moreover, a comprehensive compilation of key literature, leading journals, and influential research teams enhances the efficiency of literature retrieval and knowledge acquisition, especially for newcomers to the field. Finally, by identifying topics with low publication volume or declining attention, this study reveals critical research gaps and inspires the development of novel hypotheses and experimental designs. However, it must be acknowledged that this research has certain limitations. First, bibliometric analyses inherently rely on citation metrics, which may not always reflect the true scientific merit or innovation of a publication. Citation counts can be influenced by factors such as journal visibility, author reputation, or topic popularity, and recently published works may be underrepresented due to limited citation accumulation. Second, the data in this study were exclusively sourced from the WoSCC. Although WoSCC is a widely recognized and authoritative database, it does not index all relevant literature, potentially omitting significant studies indexed in other databases. Third, we restricted the analysis to English-language articles and reviews, which may have introduced language and publication-type biases by excluding non-English literature and other document types like conference papers or book chapters. Lastly, while quantitative methods were used to identify clusters and trends, the interpretation of co-authorship, co-citation, and keyword co-occurrence networks inevitably involved subjective judgment, which could introduce interpretation bias.

In summary, the field of tumor metabolic reprogramming has experienced rapid development over the past decade, attracting increasing attention from institutions and researchers. Targeting metabolic differences between tumor and normal cells presents a new promising anticancer strategy. Glycolysis is a current research hotspot, while metabolic reprogramming in breast cancer is an emerging and rapidly developing topic in recent years.

## Data Availability

Publicly available datasets were analyzed in this study. This data can be found here: All the data can be obtained from the Web of Science Core Collection database.
