# Supplementary material for: Frontiers and hot topics in tumor metabolic reprogramming: a bibliometric analysis from 2014 to 2023
Source: Front Oncol. 2025 Jun 24;15:1570532. doi: 10.3389/fonc.2025.1570532 (PMC12234538; doi:10.3389/fonc.2025.1570532)
Supplement: Supplementary file 1 [file Table1.docx]

Supplementary Material

# Supplementary Table

| **Supplementary Table 1. The top 10 productive journals in the field of tumor metabolic reprogramming.** | | | | | | | |
| --- | --- | --- | --- | --- | --- | --- | --- |
| **Rank** | **Source** | **Category** | **JCR**  **(2023)** | **IF**  **(2023)** | **Numbers of Publications** | **Numbers of citations** | **Average citations per publication** |
| 1 | CANCERS | ONCOLOGY | Q1 | 4.5 | 314 | 5790 | 18.44 |
| 2 | FRONTIERS IN ONCOLOGY | ONCOLOGY | Q2 | 3.5 | 308 | 6654 | 21.60 |
| 3 | INTERNATIONAL JOURNAL OF MOLECULAR SCIENCES | BIOCHEMISTRY & MOLECULAR BIOLOGY/  CHEMISTRY, MULTIDISCIPLINARY | Q1/Q2 | 4.9 | 227 | 4551 | 20.05 |
| 4 | FRONTIERS IN IMMUNOLOGY | IMMUNOLOGY | Q1 | 5.7 | 151 | 4681 | 31.00 |
| 5 | ONCOTARGET | CELL BIOLOGY/  ONCOLOGY | / | / | 133 | 5310 | 39.92 |
| 6 | CANCER RESEARCH | ONCOLOGY | Q1 | 12.5 | 120 | 5527 | 46.06 |
| 7 | CELL DEATH & DISEASE | CELL BIOLOGY | Q1 | 8.1 | 117 | 4280 | 36.58 |
| 8 | CELLS | CELL BIOLOGY | Q2 | 5.1 | 113 | 2537 | 22.45 |
| 9 | NATURE COMMUNICATIONS | MULTIDISCIPLINARY SCIENCES | Q1 | 14.7 | 112 | 6297 | 56.22 |
| 10 | SCIENTIFIC REPORTS | MULTIDISCIPLINARY SCIENCES | Q1 | 3.8 | 110 | 2956 | 26.87 |
| IF, Impact Factor; JCR, Journal Citation Reports. | | | | | | | |
